# QUALITY IMPROVEMENT IN LTC: EFFECTIVENESS OF MONTESSORI-BASED ACTIVITY PROGRAMMING IN VA COMMUNITY LIVING CENTER

**DOI:** 10.1093/geroni/igz038.3448

**Published:** 2019-11-08

**Authors:** Thomas Chacko, Kim Curyto, Michelle M Hilgeman, Virginia Keleher

**Affiliations:** 1 University at Buffalo, New York, United States; 2 Center for Integrated Healthcare, VA Western New York Healthcare System, Batavia, New York, United States; 3 Tuscaloosa VA Medical Center, Tuscaloosa, Alabama, United States; 4 Tuscaloosa VA Medical Center, Alabama, United States

## Abstract

Montessori-based Activity Programming (MAP) was adapted for Veterans Affairs (VA) Community Living Centers (CLCs) and aims to increase independence and meaningful engagement in residents with cognitive impairment. The Montessori model prioritizes offering choice, knowing and harnessing a resident’s abilities, and enabling them to carry out purposeful roles and activities. Any perceived deficit in cognitive functioning is “circumvented” by preparing the environment to support maximum independence. The implementation of MAP-VA in VA Western NY CLC involved 3 lodges, 52 staff, 16 champions, and 65 CLC residents. Standardized implementation measures demonstrated improvements over six months in five domains assessing development of a resident-directed community. Hypothesized outcomes included improved national percentile quality improvement (QI) rankings related to psychological symptoms and medications (e.g., depressive symptoms and use of antipsychotic/antianxiety medications) and physical functioning (e.g., less falls and ability to move independently). Scores six months prior to the implementation of MAP-VA (April, 2018 to September, 2018) were compared with scores during six months of implementation post training (November, 2018 to March, 2019). Compared to pre-intervention QI measures related to psychological symptoms, a clinically meaningful trajectory of symptom decrease was observed with rankings during implementation (e.g., depressive symptoms, amount of antipsychotic medications). Likewise, compared to pre-intervention QI rankings regarding physical functioning, post-training rankings showed a trajectory of improvement (e.g., help with ADLs, ability to move independently). Implementation of the MAP-VA intervention demonstrates preliminary evidence for improvement in QI measures related to psychological symptoms and physical functioning. Implications for QI efforts in VA CLCs will be presented.

